# Macrophage reprogramming through scavenger receptor-guided and cathepsin B-triggered nanodelivery: from intracellular mechanisms to translational applications

**DOI:** 10.3389/fimmu.2026.1879532

**Published:** 2026-07-10

**Authors:** Yang Huang, Le Cai, Xu Yan, Kaiyi Tong, Fei Li

**Affiliations:** 1 Department of Emergency Medicine, Central Hospital Affiliated to Shenyang Medical College, Shenyang, Liaoning, China; 2 Department of Gynecology, Shenyang Women and Child Hospital, Shenyang, Liaoning, China

**Keywords:** cathepsin B, immunometabolism, macrophage plasticity, macrophage reprogramming, nanomedicine, scavenger receptors, tumor-associated macrophages

## Abstract

Macrophages are highly plastic innate immune cells. Their functional states are dynamically shaped by inflammatory signals, metabolic stress, and disease-associated remodeling. In cancer and atherosclerosis, pathological macrophages contribute to immune suppression, plaque destabilization, and therapeutic resistance, making their reprogramming a critical translational goal. Recent advances in nanomedicine provide new opportunities to manipulate macrophage behavior by combining selective cellular entry with conditionally controlled intracellular release. This review focuses on scavenger receptor-guided and cathepsin B-triggered nanodelivery systems as a mechanism-aligned strategy for phenotypic remodeling. While scavenger receptors provide selective molecular gateways enriched in diseased macrophage populations, cathepsin B serves as an endogenous trigger for subsequent nanocarrier disassembly and payload release. We discuss how aligning targeted internalization and enzymatic release can reshape macrophage function. We also examine how these platforms engage intracellular vulnerability networks, highlighting signal transducer and activator of transcription 3 (STAT3) as a translationally relevant node that stabilizes pathological states rather than an exclusive mechanistic axis. Finally, we assess major translational challenges, including off-target sequestration, target heterogeneity, and functional bioavailability. This review aims to advance the translational development of macrophage-centered immunomodulatory therapies by linking nanodelivery design to macrophage biology and disease-relevant intracellular mechanisms,

## 1 Introduction

Macrophages are central mediators of innate immunity and play essential roles in host defense, tissue homeostasis, and repair (1). They display remarkable phenotypic plasticity and can adopt diverse functional states in response to dynamic microenvironmental signals (1–3). Although the classical M1/M2 classification does not fully encompass macrophage heterogeneity *in vivo* (1, 3), it remains a useful conceptual model for describing two opposite ends of a broader activation spectrum (1). Classically activated M1-like macrophages are generally characterized by pro-inflammatory, antimicrobial, and often antitumor activities. In contrast, alternatively activated M2-like macrophages are more commonly associated with anti-inflammatory responses, tissue remodeling and fibrosis. In atherosclerotic plaques, macrophages more commonly display inflammatory and lipid-loaded features that overlap with M1-like activation and contribute to foam-cell formation, persistent inflammation, and plaque progression (4, 5). In solid tumors, tumor-associated macrophages (TAMs) are frequently shaped by the tumor microenvironment (TME) toward M2-like, immunosuppressive, and tissue-remodeling programs (6–8). TAMs promote tumor growth, invasion, metastasis, and angiogenesis (6–8).toward They also contribute to resistance to chemotherapy and immunotherapy through the secretion of extracellular matrix-remodeling enzymes, angiogenic mediators, and immunosuppressive cytokines (7, 9, 10). Macrophages in both cancer and atherosclerosis undergo profound alterations in lipid metabolism, lysosomal processing, and stress adaptation (5, 11, 12). This shared immunometabolic remodeling provides a cross-disease rationale for macrophage-targeted therapeutic intervention. Accordingly, the reprogramming of pathological macrophages into therapeutically favorable states has emerged as a major focus of modern therapeutic development (4, 13).

Recent advances in nanomedicine have created new opportunities for the precise modulation of macrophage phenotype and function (14, 15). Compared with free drugs, nanocarriers offer tunable physicochemical properties, improved pharmacokinetics, enhanced cargo-loading capacity, and versatile surface modification strategies (15, 16). Nanoparticles may accumulate in pathological tissues through the enhanced permeability and retention (EPR) effect in some solid tumors or through increased vascular permeability in inflamed lesions. However, passive accumulation is heterogeneous and is often insufficient to ensure macrophage-selective delivery. Active targeting strategies use ligand–receptor interactions to improve cellular internalization by selected macrophage populations. Stimulus-responsive systems can further restrict cargo release to disease-relevant intracellular compartments. Nanomedicine enables the rational integration of two particularly relevant design principles: receptor-mediated active targeting and microenvironment-responsive intracellular release (14, 17). Together, these features may enhance delivery to disease-associated macrophages while limiting premature drug exposure in non-target tissues (15).

Among the macrophage-associated surface molecules explored for active targeting, scavenger receptors have attracted considerable attention because they are enriched in pathological macrophage populations, including M2-like TAMs and lipid-laden macrophages (5, 18, 19). Receptors such as cluster of differentiation 36 (CD36), scavenger receptor class A member 1/macrophage scavenger receptor 1 (SR-A1/MSR1), macrophage receptor with collagenous structure (MARCO), and scavenger receptor class B type 1 (SR-B1) participate not only in ligand internalization but also in lipid metabolism, inflammatory signaling, phagocytosis, and immunoregulatory adaptation (19–21). In atherosclerosis, scavenger receptor-mediated internalization of oxidized lipids reinforces lipid stress and inflammatory signaling (22, 23). In tumors, altered lipid acquisition and metabolic rewiring may support the persistence of immunosuppressive macrophage states (18, 24, 25). Thus, scavenger receptors are not merely molecular docking sites for nanocarriers, but functionally relevant participants in pathological macrophage biology (19, 20).

Cathepsin B (CTSB), a lysosomal cysteine protease commonly elevated in tumors, inflammatory lesions, and macrophage-rich pathological niches (26), has emerged as an attractive endogenous trigger for smart nanocarrier disassembly and controlled intracellular drug release (26, 27). CTSB-triggered systems are especially relevant in protease-rich and lysosomally active lesions (28), where they can exploit disease-related intracellular processing pathways to improve spatiotemporal control of payload release. The combination of scavenger receptor-mediated internalization and CTSB-triggered release therefore provides a compelling design logic for macrophage-directed nanotherapy (28).

**Figure 1:**
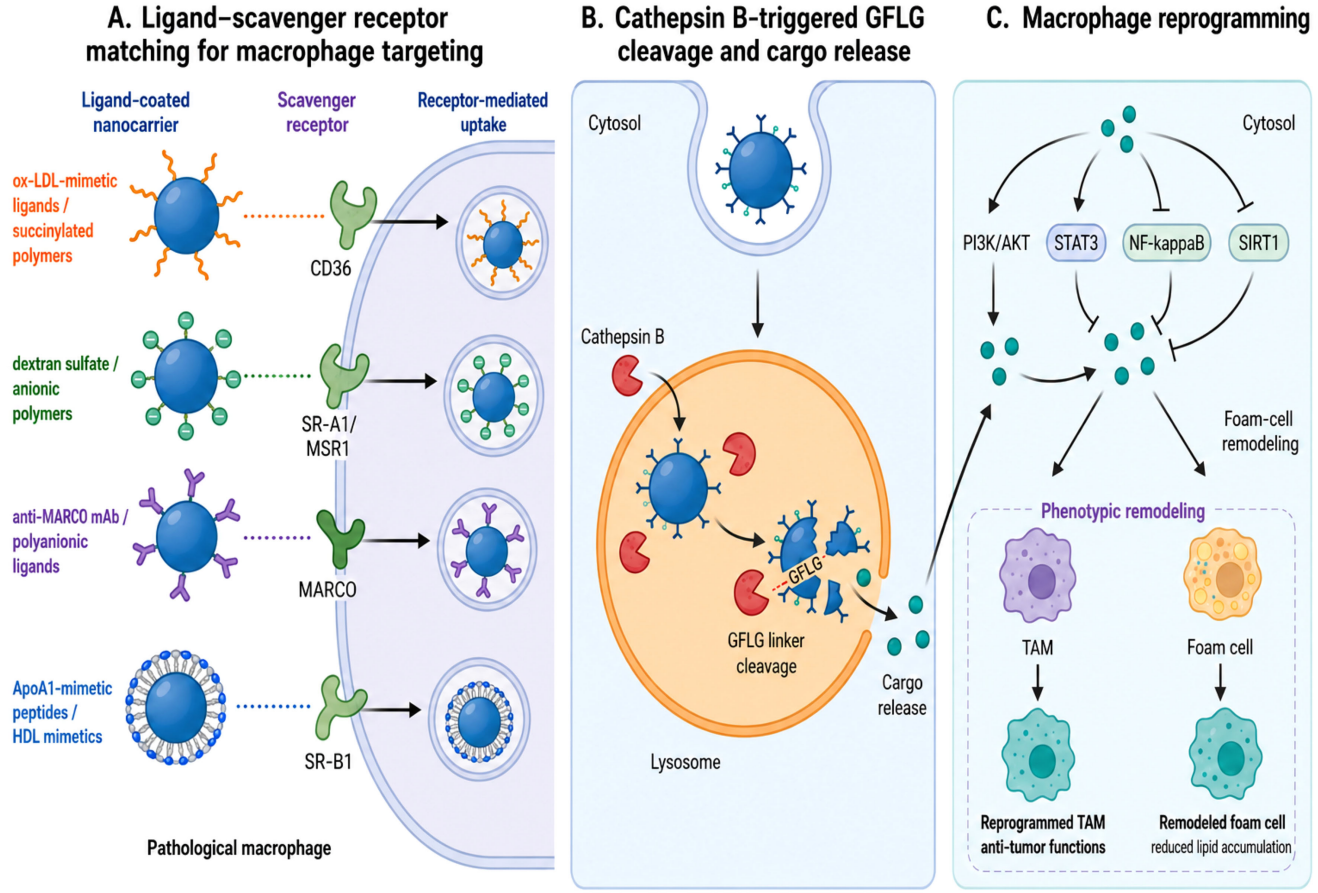
Mechanism-aligned macrophage reprogramming through scavenger receptor-guided internalization and cathepsin B-triggered nanodelivery. **(A)** Representative ligand–scavenger receptor matching strategies for macrophage targeting. Ligand-coated nanocarriers may engage scavenger receptors through examples such as ox-LDL-mimetic ligands or succinylated polymers/CD36, dextran sulfate or anionic polymers/SR-A1/MSR1, anti-MARCO monoclonal antibody or polyanionic ligands/MARCO, and ApoA1-mimetic peptides or HDL mimetics/SR-B1. These ligand–receptor interactions support receptor-mediated uptake into pathological macrophages. **(B)** After internalization, nanocarriers traffic into endosomal/lysosomal compartments, where cathepsin B cleaves cathepsin B-sensitive linkers such as Gly-Phe-Leu-Gly (GFLG), leading to nanocarrier disassembly and cargo release. **(C)** Released cargo can modulate intracellular vulnerability networks, including PI3K/AKT, STAT3, NF-kappaB, and SIRT1. These pathway-directed effects may promote TAM reprogramming and foam-cell remodeling, thereby shifting pathological macrophages toward more therapeutically favorable states. Created in BioRender. Huang, (Y) (2026) https://BioRender.com/9p8chcc. ApoA1, apolipoprotein A1; CD36, cluster of differentiation 36; GFLG, Gly-Phe-Leu-Gly; HDL, high-density lipoprotein; MARCO, macrophage receptor with collagenous structure; mAb, monoclonal antibody; MSR1, macrophage scavenger receptor 1; NF-kappaB, nuclear factor-kappa B; ox-LDL, oxidized low-density lipoprotein; PI3K/AKT, phosphoinositide 3-kinase/protein kinase B; SR-A1, scavenger receptor class A member 1; SR-B1, scavenger receptor class B type 1; STAT3, signal transducer and activator of transcription 3; SIRT1, sirtuin 1; TAM, tumor-associated macrophage.

Macrophage-targeted nanotherapy should be viewed not merely as a delivery approach, but as a mechanistically informed strategy for reshaping disease-associated macrophage states (15, 29). In this review, nanomedicine is taken as the main organizing framework to summarize recent progress in scavenger receptor-targeted (30, 31) and CTSB-triggered (27, 28, 32) nanosystems for macrophage phenotypic remodeling. We focus on how these platforms can be engineered to achieve selective macrophage internalization (33, 34), controlled intracellular release (35), and functional reprogramming in cancer and atherosclerosis (36, 37). Particular attention is given to intracellular vulnerability networks (38) that determine whether selective delivery can be translated into durable macrophage remodeling. In addition, we discuss the contribution of disease-stabilizing signaling and transcriptional programs, with signal transducer and activator of transcription 3 (STAT3) considered as a representative node within a broader intracellular network framework (39–41). The overall mechanism of scavenger receptor-guided internalization, CTSB-triggered release, and pathway-directed macrophage remodeling is summarized in **Figure 1**. We aim to highlight both the therapeutic promise and the translational challenges of macrophage-centered precision nanomedicine (42).

## 2 Macrophage scavenger receptors: molecular gateways for precision nanodelivery

Scavenger receptors are a diverse group of pattern recognition receptors expressed on macrophages, dendritic cells, endothelial cells, and several other cell types (19, 43). They were initially identified for their capacity to bind and internalize modified lipoproteins, such as oxidized low-density lipoprotein (ox-LDL) and acetylated low-density lipoprotein (acetylated LDL) (23, 44). Studies have shown that scavenger receptors participate in a broad range of biological processes, including pathogen clearance, apoptotic cell engulfment, lipid metabolism, inflammatory signaling, and tissue remodeling (19, 43). Because of these roles, scavenger receptors are highly relevant to both disease pathogenesis and targeted drug delivery (19, 44).

Based on structural features, scavenger receptors are classified into multiple subfamilies (45). Among these, the subtypes most relevant to nanomedicine are SR-A1/MSR1, CD36, MARCO, and SR-B1 (19, 30, 45). These receptors differ in ligand preference, signaling behavior, and cellular distribution (19). Despite these differences, they share a feature of translational importance: their marked upregulation in pathological macrophage subsets (19, 23). Within tumors and atherosclerotic plaques, this receptor overexpression reflects the cells’ metabolic adaptation to local microenvironmental stress, rendering these scavenger receptors promising molecular gateways for selective nanodrug delivery (19, 23, 30).

### 2.1 Heterogeneous expression and functional regulation of scavenger receptors in pathological microenvironments

The expression pattern of scavenger receptors is highly context-dependent and varies considerably across disease models, tissue sites, and macrophage activation states (46, 47). In atherosclerosis, endothelial dysfunction and local inflammatory signaling promote monocyte recruitment into the arterial intima, where these cells differentiate into macrophages and progressively upregulate receptors such as SR-A1 and CD36 (48–50). These receptors facilitate the internalization of modified lipoproteins, especially ox-LDL, resulting in intracellular lipid accumulation and foam cell formation (47, 49, 51). Ligand binding to these receptors may also activate intracellular signaling pathways that amplify inflammation and promote lesion progression (48, 49).

CD36 is a representative example of this dual role (20, 52). In addition to mediating ox-LDL uptake, CD36 can cooperate with Toll-like receptor 4 (TLR4) and Toll-like receptor 6 (TLR6) complexes to activate nuclear factor kappa B (NF-kappaB) and related kinase pathways (53, 54). Through this mechanism, lipid uptake and inflammatory amplification become functionally coupled (52). CD36 is not only a marker of diseased macrophages but also an active participant in the pathological circuitry that sustains chronic inflammation (20, 48). Similar principles apply to TAMs (18, 19). Within the TME, TAMs undergo metabolic rewiring and increase their acquisition of fatty acids and other lipid species (18, 25). Scavenger receptors such as CD36 and SR-B1 contribute to this process, allowing TAMs to adapt to nutrient-deprived conditions and support sustained mitochondrial metabolism (52, 55). This lipid influx can in turn influence macrophage polarization through signaling pathways involving peroxisome proliferator-activated receptor gamma (PPARγ), adenosine monophosphate-activated protein kinase (AMPK), and related metabolic regulators, thereby reinforcing M2-like and immunosuppressive states (25, 56, 57). In this sense, scavenger receptors function as both metabolic sensors and immune modulators (20, 52).

These observations have important implications for nanomedicine (58–60). Targeting scavenger receptors may improve intracellular drug delivery to pathological macrophages (61, 62), but it may also interfere with receptor-dependent lipid uptake and signaling networks (31). Therefore, scavenger receptor-targeted nanoplatforms may exert dual effects: they can serve as efficient delivery vehicles while simultaneously modulating pathogenic receptor activity (60, 63). This dual role makes scavenger receptors especially relevant therapeutic entry points in diseases where receptor activity contributes directly to pathology (64).

This ligand internalization directly stabilizes stress-adapted macrophage phenotypes (20). In atherosclerosis, receptors such as CD36 and SR-A1 facilitate the uptake of modified lipids, amplifying metabolic stress, driving foam cell formation, and reinforcing inflammatory signaling (23, 36). In tumors, altered lipid uptake through scavenger receptors enables TAMs to endure nutrient deprivation, supporting the metabolic rewiring necessary for maintaining immunosuppressive programming (18, 19). These observations establish that scavenger receptors are not only molecular gateways for nanodelivery, but also functional participants in the immunometabolic remodeling that supports tumor progression and atherosclerotic plaque evolution (19, 20).

The biological effects of scavenger receptor-targeted nanoplatforms may extend beyond simple payload delivery (14–16, 19). Because scavenger receptors are functionally linked to lipid uptake, phagocytosis, inflammatory signaling, and metabolic adaptation, ligand-decorated nanoparticles may influence macrophage behavior through cargo-independent mechanisms (19–21, 52). One possible mechanism is competitive ligand binding. Nanocarriers that engage CD36 or MSR1 may reduce the access of endogenous ligands such as ox-LDL, thereby attenuating lipid influx, lipid-associated stress, and foam-cell formation in atherosclerotic macrophages (20, 60, 63). Another mechanism is receptor-mediated signaling modulation. Multivalent receptor engagement may promote receptor clustering, depending on ligand identity, ligand density, and nanocarrier architecture (19, 31, 52). Such clustering may alter downstream pathways, including CD36/TLR4/TLR6-associated NF-kappaB signaling and MSR1-associated JNK activation (21, 52, 54). MARCO-directed targeting strategies may also influence TAM function through receptor engagement and macrophage-associated immunomodulatory effects (19, 42). Therefore, scavenger receptor-guided nanocarriers should be evaluated as both intracellular delivery vehicles and receptor-engaging immunomodulatory platforms (14, 15). Their biological effects may arise from a dynamic interplay between cargo-dependent and cargo-independent mechanisms (14, 15, 19, 20).

### 2.2 Nanomedicine strategies targeting CD36 and SR-B1 for lipid metabolism intervention

Among the scavenger receptors implicated in pathological lipid handling, CD36 and SR-B1 are especially promising for nanomedicine-based intervention (59, 65). Both receptors are closely involved in lipid transport and are frequently dysregulated in diseases characterized by aberrant macrophage lipid metabolism, including atherosclerosis and cancer (59, 62).

A number of nanoplatforms have been designed to exploit the lipid-recognition properties of CD36 (59, 66). One strategy is to construct biomimetic nanoparticles that mimic modified lipoproteins in charge distribution and hydrophobicity, thereby enabling competitive interaction with CD36 (66, 67). Such systems may reduce the accumulation of endogenous ox-LDL while simultaneously delivering therapeutic cargo to lipid-laden macrophages (59, 62). For example, amphiphilic nanoparticles can be designed to replicate key physicochemical features of ox-LDL, allowing them to inhibit pathological lipid influx (67). After cellular entry, these platforms may release therapeutic agents that attenuate lipid-associated stress and macrophage dysfunction (62). This design is appealing because it targets both the upstream driver of foam-cell formation and downstream inflammatory or metabolic consequences (60).

Beyond mimicking lipids, researchers have utilized specific antibodies to directly target CD36 (62, 68). Anti-CD36 mesoporous silica nanoparticles and CD36-binding lipid-mimetic nanoparticles have been used to improve macrophage or foam-cell targeting in atherosclerotic lesions (62, 68). In this context, the nanoparticle not only improves delivery efficiency but also enhances lesion-specific accumulation (62). These nanocarriers often deliver drugs designed to promote cholesterol efflux or restore metabolic balance. By directing these agents specifically to foam cells, the systems may reverse the diseased phenotype and resolve plaque inflammation. SR-B1 is another highly relevant target because of its physiological role as a receptor for high-density lipoprotein (HDL). This feature has inspired the development of reconstituted HDL (rHDL) nanoparticles and HDL-mimetic nanocarriers (65, 69–71). By incorporating apolipoprotein A-I (ApoA1) or ApoA1-mimetic peptides, researchers have generated biomimetic systems capable of engaging SR-B1 and delivering therapeutic payloads into macrophages and tumor cells (69, 70). These nanoparticles are particularly well suited to this purpose because they exploit a natural lipid transport pathway, which may facilitate efficient cellular entry and favorable biocompatibility (69).

In cancer nanomedicine, SR-B1-targeted systems have been explored for the depletion or reprogramming of immunosuppressive TAMs (19, 72). For example, lipid nanoparticles co-functionalized with ApoA1-mimetic peptides and M2 macrophage-targeting ligands have been used to deliver small interfering RNA (siRNA) against macrophage survival or polarization-related targets (42, 73, 74). Such platforms can promote selective uptake by TAMs and reduce the abundance of pathogenic macrophage subsets, thereby indirectly suppressing tumor growth (72, 73). These studies demonstrate that biomimetic targeting of endogenous lipid trafficking pathways provides a practical route for macrophage-directed therapy (19, 72).

Taken together, CD36- and SR-B1-oriented nanomedicine strategies illustrate how receptor biology can be integrated with metabolic intervention (19, 59). Rather than merely treating these receptors as passive docking sites, well-designed nanocarriers can exploit their pathological functions to achieve both precise delivery and active therapeutic reprogramming (72, 73).

### 2.3 MSR1- and MARCO-mediated selective targeting of TAMs

Beyond CD36 and SR-B1, MSR1 and MARCO are important targets for TAM-directed nanomedicine (75). Both receptors are frequently enriched in macrophage populations residing in tumor tissue and have been associated with immunoregulatory and tumor-promoting functions in several solid malignancies (19).

MARCO has been reported to be highly expressed on TAMs in cancers such as breast cancer and hepatocellular carcinoma (76), where its abundance is often associated with poor clinical outcome (76, 77). This has prompted the development of MARCO-directed antibodies as both functional modulators and targeting ligands (78, 79). Antibody-based engagement of MARCO may not only improve nanoparticle accumulation in TAMs but also alter macrophage behavior through Fc receptor-dependent immune effects (79, 80). In other words, MARCO-targeted systems may combine delivery and immunomodulation in a single platform (79).

MSR1 is another important target for macrophage-directed nanodelivery (75). It naturally binds to a wide array of negatively charged molecules (81). Capitalizing on this feature, researchers frequently use negatively charged polymers, such as dextran sulfate (81, 82) or hyaluronic acid (83, 84), to drive preferential nanoparticle binding to MSR1-rich macrophages (75, 85). This electrostatic targeting principle has proven useful for the development of macrophage-selective nanocarriers, particularly in tumor and inflammatory settings where MSR1 expression is enhanced (75, 82).

MSR1 is not merely a passive internalization receptor. Its engagement may trigger intracellular signaling and thereby influence macrophage phenotype (21). This signaling raises an important design concern, as MSR1-targeted nanocarriers may inadvertently amplify pro-inflammatory signaling (21, 85). The biological consequence of this effect is likely to be disease dependent (19, 85). In TAMs, controlled inflammatory reactivation may support antitumor macrophage reprogramming (13, 75). In atherosclerotic plaques, however, excessive JNK-associated inflammatory activation may aggravate local inflammation and increase plaque instability risk (4, 5, 21). Several strategies may help reduce this risk. Ligand density, binding avidity, particle size, surface charge, and ligand orientation should be carefully optimized to avoid excessive receptor clustering (14, 31). Shielding strategies or cleavable surface architectures may reduce unintended receptor activation before lesion accumulation (14, 17). MSR1-targeted platforms can be paired with anti-inflammatory or pathway-modulating cargo to counterbalance unwanted inflammatory signaling after intracellular release (15, 82, 83). Therefore, comprehensive evaluation of these systems should integrate macrophage uptake and receptor activation with downstream analyses of JNK signaling, cytokine production, and disease-relevant phenotype conversion (15, 21, 85).

In interleukin-4 (IL-4)-polarized macrophages, MSR1 ligation has been reported to induce lysine 63 (K63)-linked polyubiquitination (21, 86). This event promotes recruitment of the transforming growth factor-beta-activated kinase 1/mitogen-activated protein kinase kinase 7/c-Jun N-terminal kinase (TAK1/MKK7/JNK) signaling complex to phagosomal compartments (21), leading to JNK activation and increased expression of pro-inflammatory mediators (86, 87). This observation suggests that receptor engagement may contribute to phenotypic reprogramming under certain conditions and raises the possibility that a properly designed receptor-binding nanoplatform could exert synergistic biological effects: facilitating intracellular drug delivery while also modulating the signaling consequences of receptor activation (21).

A variety of MSR1-targeted nanostructures have been explored for TAM reprogramming (75, 82). Nanoparticles coated with hyaluronic acid (84, 88), dextran derivatives (75, 82), or receptor-specific antibodies (80) can improve the delivery of chemotherapeutics, nucleic acids, or immunomodulatory agents into TAMs. When these nanocarriers are loaded with cargos such as vascular endothelial growth factor (VEGF)-targeting siRNA (42, 89), pro-inflammatory immune agonists (90), or metabolism-modifying compounds, they may shift TAMs away from suppressive phenotypes and improve anti-tumor immunity. Therefore, MSR1 and MARCO represent more than surface markers of pathological macrophages; they are functionally engaged receptors with meaningful translational potential (19, 80).

Overall, scavenger receptors provide a robust molecular interface between macrophage pathobiology and nanotherapeutic design (19, 75). These receptors exhibit variable, lesion-specific expression patterns while governing lipid metabolism and immune regulation. This dual role supports the development of nanoplatforms capable of both selective cellular entry and functional intervention. In the following section, we further examine how intracellular proteolytic pathways, particularly those involving CTSB, can be leveraged to enable spatiotemporally controlled drug release after receptor-guided uptake.

## 3 CTSB-triggered nanodelivery systems: spatiotemporally controlled release switches

Nanotherapeutic systems that enter macrophages through receptor-guided uptake must pass through the endo/lysosomal compartment, which represents a major intracellular bottleneck for effective cargo delivery (91). Many therapeutic cargos may undergo degradation or become functionally trapped if they fail to escape or be released after endocytosis. Rather than treating the lysosomal compartment as an obstacle, recent advances in smart nanomedicine have sought to exploit its biochemical properties as an opportunity for controlled intracellular release (26). Among the endogenous triggers available in pathological tissues and intracellular vesicles, CTSB has emerged as a widely used and mechanistically tractable proteolytic trigger (26). This design is supported by the pathological enrichment of CTSB in lysosomally active macrophage-rich lesions and by its capacity to cleave rationally engineered peptide linkers after intracellular trafficking (92, 93).

### 3.1 Pathological enrichment and lysosomal catalytic features of CTSB

CTSB is predominantly localized within lysosomes under physiological conditions, where it contributes to protein turnover, autophagy, antigen processing, and maintenance of intracellular homeostasis (26, 94). Its activity is tightly regulated by lysosomal pH and endogenous inhibitors such as cystatins (94, 95). Under normal conditions, this regulatory balance prevents excessive proteolysis and protects cellular integrity. Under pathological conditions, CTSB expression and activity are frequently increased (26, 92). This increase in CTSB expression and activity may result from several converging microenvironmental factors, including hypoxia, metabolic stress, inflammatory cytokine signaling, and altered lysosomal dynamics (94, 96). M2-like macrophages and TAMs often exhibit enhanced lysosomal activity, allowing CTSB to participate in broader proteolytic and tissue-remodeling programs (92, 96).

For nanodelivery design, one of the most important features of CTSB is pH-dependent catalytic behavior (97, 98). CTSB exhibits strong activity in the mildly acidic environments of late endosomes and lysosomes, typically around pH 4.5–5.5 (94, 98). Its catalytic efficiency is substantially reduced in the neutral pH of blood circulation and most normal extracellular tissues (91, 97). This sharp contrast in activity creates a favorable biochemical basis for designing nanocarriers that remain stable during systemic circulation but undergo cleavage after internalization into pathological cells (26, 91). Another key advantage is that CTSB abundance and activity often correlate more closely with pathological state than with a specific cell lineage (26, 94). Rather than treating CTSB as a strictly phenotype specific biomarker, it is more accurate to regard it as a pathologically enriched intracellular trigger that is particularly useful in disease contexts where macrophages play a major pathogenic role (94, 99).

### 3.2 Gly-Phe-Leu-Gly-based CTSB-cleavable nanostructures: design principles and assembly logic

CTSB-triggered nanocarrier design has largely relied on peptide sequences that can be selectively recognized and cleaved by CTSB (26). Among these, GFLG has become one of the most widely used and well-characterized CTSB-sensitive linkers (100, 101). GFLG combines relative stability in circulation with efficient cleavage in lysosomal compartments (101, 102) and has therefore been incorporated into a broad range of nanostructures, including polymer-drug conjugates, micelles, liposomes, dendrimers, and self-assembled peptide systems (100–103).

In a typical modular design, the GFLG sequence is inserted between the carrier scaffold and the therapeutic cargo (93, 101). Once the nanocarrier has undergone receptor-guided uptake and trafficked into endo/lysosomal compartments, CTSB recognizes and cleaves the peptide linker, thereby releasing the drug or destabilizing the carrier structure (93, 100, 104). The outcome of GFLG cleavage depends on the formulation strategy. In conjugate-based systems, CTSB-mediated cleavage releases a linked cytotoxic drug or signaling inhibitor (93, 101). In assembly-based systems, cleavage can destabilize the carrier and accelerate payload release through structural collapse or morphological transformation (103, 104). In self-assembled or amphiphilic systems, CTSB-mediated removal of a hydrophilic peptide segment can alter the balance between hydrophilic and hydrophobic interactions, leading to secondary assembly behavior or shape transformation (100, 104). For example, a spherical micelle may rearrange into elongated or fibrous structures after enzymatic cleavage, thereby prolonging retention at the target site or within the cell (104). Such enzyme-triggered morphological transitions are especially useful because they provide an additional level of control over drug exposure and intracellular fate (103, 104).

Similarly, in polymeric delivery platforms, CTSB-sensitive peptides can be inserted into the backbone of amphiphilic block copolymers or between functional segments of the material (101, 105). Once exposed to the lysosomal environment, cleavage of these peptide domains may lead to carrier disassembly and accelerated release of hydrophobic drugs, immunomodulators, or pathway inhibitors (101, 105). In macrophage-targeted nanomedicine, this strategy is especially relevant because receptor-guided uptake through scavenger receptors naturally directs nanoparticles into the endo/lysosomal pathway (32, 106).

In translational terms, GFLG-based systems offer several advantages. They rely on endogenous pathological protease activity rather than external stimuli, which may simplify treatment logistics (100, 101). They can also be integrated into a wide variety of carrier chemistries without fundamentally altering the targeting architecture (100, 101). In addition, they allow the design of conditionally activated release profiles that are potentially more selective than passive intracellular diffusion (100). However, the success of these systems depends on several variables, including linker accessibility, local CTSB abundance, intracellular trafficking kinetics, and competition from other proteases (107, 108). Although GFLG remains a broadly used and well-validated CTSB-sensitive motif (101), its performance should be interpreted in a context-dependent manner rather than assumed to be universally optimal.

### 3.3 Cooperative delivery strategies integrating CTSB with additional microenvironmental triggers

Although CTSB-triggered systems have shown strong potential (26), reliance on a single enzymatic trigger may not provide sufficient selectivity or robustness in complex pathological tissues (27). Because CTSB activity may vary across lesions and disease stages (94), single-trigger systems may show inconsistent activation in heterogeneous tissues. This limitation has driven the development of dual- or multi-trigger nanocarriers that combine CTSB sensitivity with other disease-associated stimuli, such as acidic pH, reactive oxygen species (ROS), glutathione-sensitive linkages, or hypoxia (27).

Integrating pH sensitivity with CTSB recognition matches the natural pathway of macrophage endocytosis (91, 109). Nanocarriers can be engineered to exploit the acidic endosomal environment to undergo initial structural loosening or charge reversal, thereby exposing the CTSB-cleavable domain (110). This cascade-like sequence supports rapid payload release once the carrier reaches protease-rich lysosomes (109). ROS/CTSB-cooperative systems represent another design category, particularly in highly oxidative inflammatory lesions and atherosclerotic plaques (32, 111). In these formulations, an ROS-sensitive outer layer may degrade under local oxidative conditions, exposing an inner CTSB-sensitive core and thereby improving lesion selectivity while reducing premature activation during circulation (111).

The main advantage of multi-trigger systems lies in their ability to increase conditional specificity (26, 27). Instead of relying on a single pathological parameter, they require the convergence of two or more disease-associated features (110, 111). This logic may be especially valuable in diseases characterized by substantial biological heterogeneity, such as pancreatic cancer, glioblastoma (28), or advanced atherosclerosis (32). However, increasing the number of responsive modules also increases formulation complexity and may complicate manufacturing, stability assessment, and reproducibility (27, 112). The translational value of multi-trigger platforms should be judged by whether the added complexity produces a measurable improvement in selectivity, intracellular release, or therapeutic efficacy. In this sense, cathepsin B-triggered systems are best viewed as programmable intracellular release modules that connect receptor-guided uptake with disease-associated lysosomal processing.

## 4 Intracellular vulnerability networks underlying nanocarrier-mediated macrophage phenotypic remodeling

Selective macrophage entry and CTSB-triggered cargo release do not necessarily guarantee durable phenotypic reprogramming (113). Stable remodeling of macrophage function depends on whether the released cargos engage intracellular vulnerability networks (114, 115). These networks regulate inflammatory tone, metabolic adaptation, lysosomal processing, and transcriptional stabilization (22, 116, 117). In pathological settings such as cancer and atherosclerosis, macrophage phenotypes are maintained by interconnected regulatory layers involving lipid handling, stress adaptation, cytokine signaling, lysosomal remodeling, and epigenetic control (5, 117, 118). Rather than being driven by a single master regulator, macrophage phenotypic remodeling is better understood as a coordinated process shaped by multiple druggable nodes (115, 119). In this framework, STAT3 is emphasized as a representative and translationally relevant node, but it should be interpreted within a broader network context rather than as the sole determinant of macrophage fate (113, 120, 121).

### 4.1 Metabolic, lysosomal, and stress-associated determinants of macrophage state transitions

Macrophage polarization is closely linked to intracellular metabolic organization (36, 122, 123). M1-like macrophages are often characterized by enhanced glycolysis and rapid inflammatory output, whereas M2-like macrophages more commonly rely on oxidative phosphorylation, fatty acid oxidation, and sustained mitochondrial metabolism (122, 123). Although this dichotomy is context-dependent and incomplete, it provides a useful framework for understanding how metabolic states support distinct macrophage functions (123). In pathological microenvironments, macrophages are simultaneously exposed to altered lipid availability, hypoxia, inflammatory cytokines, and oxidative stress (22, 122). These cues reshape not only energy metabolism but also chromatin accessibility, transcription factor activity, and lysosomal demand (122). Scavenger receptors such as CD36, SR-B1, MSR1, and MARCO are important because they link extracellular ligand internalization to intracellular metabolic and stress adaptation (19, 20, 124). This continuous receptor-mediated influx may increase lysosomal burden, perturb mitochondrial function, and amplify stress-sensitive signaling (20, 125).

Lysosomal remodeling is especially important in this context (92, 125). Because receptor-mediated endocytosis routes many nanocarriers into endo/lysosomal compartments, the phenotypic impact of nanomedicine depends in part on how pathological macrophages process internalized material (91, 125). CTSB-rich lysosomes therefore represent more than mere trigger sites for drug release (91). They indicate a broader, stress-adapted intracellular state defined by proteolytic activation and altered trafficking, rendering these macrophages responsive to phenotype-modulating interventions (91, 125).

Taken together, macrophage state transitions are shaped by the coordinated interaction of metabolic rewiring, lysosomal processing, and stress-associated adaptation (22, 55, 126). These intertwined pathways dictate whether selectively delivered cargos will successfully dismantle or reinforce pathological macrophage phenotypes (36, 125, 127).

### 4.2 Druggable intracellular signaling and transcriptional networks in macrophage reprogramming

Although pathological macrophage phenotypes are sustained by complex and overlapping regulatory mechanisms, several intracellular signaling and transcriptional networks emerge as therapeutically tractable targets (113, 128). These include cytokine-responsive transcriptional hubs, inflammatory signaling circuits, and metabolic regulators that govern macrophage survival, polarization, and functional persistence (29, 36). Among the most relevant pathway classes are phosphoinositide 3-kinase/protein kinase B (PI3K/AKT)-associated polarization programs (113, 129), NF-kappaB related inflammatory signaling pathways (113, 129), and transcriptional regulators such as STAT3 (39, 113). In addition, metabolic modulators including sirtuin 1 (SIRT1) and related stress-adaptive regulators may influence how macrophages respond to lipid burden and inflammatory cues (36). The translational relevance of these nodes lies in the fact that they can be engaged by small molecules, nucleic acids, or combinatorial nanocarrier payloads following targeted intracellular delivery (76, 88).

In practice, macrophage-directed nanocarriers do not act through a singular mechanistic axis (130). They may interfere with inflammatory transcription, suppress immunosuppressive signaling, restore metabolic balance, or destabilize phenotype-maintaining circuits depending on the disease context and therapeutic cargo (29). Accordingly, the value of scavenger receptor-targeted and CTSB-triggered systems lies not only in selective uptake and conditional release, but also in their capacity to engage different intracellular vulnerability networks (88).

Within this broader intracellular vulnerability network, STAT3 serves as a representative transcriptional node. It bridges cytokine-derived signals with downstream programs that support macrophage persistence, immunoregulation, and tissue remodeling (39, 113, 120). However, STAT3 should not be viewed as the only actionable target. Rather, it provides a clear example of how pathway-modulating cargos can be matched with receptor-guided uptake and intracellular release in macrophage-centered nanomedicine (40, 88).

### 4.3 STAT3 as a representative node linking cytokine signaling, lysosomal stress, and phenotype persistence

STAT3 is an important mediator of cytokine-driven transcriptional responses in macrophages (113, 120). In disease-associated macrophages, sustained STAT3 activity can support immunoregulatory, tissue-remodeling, and tumor-supportive programs (39, 121, 131). In TAMs, persistent STAT3 activation promotes immune tolerance, extracellular matrix remodeling, angiogenic support, and resistance to spontaneous repolarization (38, 77, 120). These features make STAT3 a practical mechanistic reference point for macrophage reprogramming because it is biologically relevant, druggable, and compatible with multiple nanomedicine-based intervention strategies (39, 40).modelingprogrammodelingSTAT3 should not be interpreted in isolation. Its activity is shaped by the broader intracellular environment and stress-adapted states. In pathological macrophages with enhanced lysosomal activation and proteolytic burden, CTSB-rich intracellular states may coexist with the kinds oy and stress-associated conditions that also favor STAT3-dependent phenotype persistence (96, 132, 133). While this does not establish a universally linear CTSB–STAT3 signaling axis, it supports the idea that lysosomal proteolytic state and STAT3-centered transcriptional stabilization may converge within the same disease-associated macrophage programs (132). This distinction is important for nanomedicine because STAT3 inhibition is most valuable when it is aligned with selective macrophage entry, intracellular release, and disease-relevant pathway engagement (38, 39). The key point is not that STAT3 is the only meaningful target, but that it serves as a representative node linking persistent intracellular signaling to therapeutically actionable phenotype remodeling (134).

### 4.4 Translational implications: nanodelivery of pathway-modulating cargos for macrophage reprogramming

The translational value of the intracellular vulnerability network concept lies in its implications for payload selection. After scavenger receptor-mediated internalization and CTSB-triggered release, therapeutic efficacy depends on whether the released cargo engages an intracellular target that is relevant to macrophage phenotype maintenance (88). Current nanodelivery strategies for macrophage remodeling already reflect this logic. For example, some platforms deliver inhibitors of PI3K/AKT-associated signaling to interfere with M2-like polarization and macrophage-mediated tumor support (42, 88). Others employ nucleic acid cargos, such as siRNA or miRNA, to dampen inflammatory amplification circuits or destabilize transcriptional programs associated with disease-promoting macrophage states (39, 135). Still others use metabolic modulators to restore intracellular homeostasis in foam cells or inflammatory macrophages (30, 36). Taken together, these examples show that translational nanomedicine for macrophage reprogramming can be viewed as targeted intracellular intervention.

STAT3-targeting cargos provide one example of this mechanism-matched strategy. Small-molecule inhibitors, siRNA, antisense oligonucleotides, and decoy oligonucleotides can be useful only when they are delivered to the appropriate macrophage population and reach the relevant cytoplasmic or nuclear machinery (39, 40, 135). Thus, the therapeutic value of pathway-modulating nanocarriers lies not simply in inhibiting a selected target, but in matching the uptake route, release mechanism, and payload action to the disease-maintaining macrophage program (77, 136). Therefore, the most promising future direction is not the exclusive prioritization of one pathway over all others, but the rational alignment of three elements: a disease-relevant uptake route, a reliable intracellular release trigger, and a mechanistically matched cargo (15, 137). In this sense, receptor-targeted and CTSB-triggered nanocarriers provide a platform for engaging multiple intracellular vulnerability networks through mechanism-matched payload design (28, 32).

## 5 Translational applications of mechanism-aligned macrophage reprogramming in major diseases

The translational value of macrophage-targeted nanomedicine requires clear distinction between design principles and disease-specific formulation. Cancer and atherosclerosis both contain macrophage-rich pathological microenvironments. Within these distinct niches, dysregulated scavenger receptor expression, hyperactivated lysosomal processing, and intense metabolic stress offer vulnerabilities for targeted intervention. The same nanoparticle configuration is unlikely to be equally effective in both diseases. Receptor-guided internalization and CTSB-triggered release should therefore be viewed as adaptable design principles rather than a universal formulation strategy.

In tumors, nanocarriers must address stromal barriers and target heterogeneous TAM populations without compromising systemic chemotherapy or immunotherapy. In glioblastoma, these delivery constraints are further amplified by anatomical blood-brain barriers. Payloads in these settings are typically designed to reduce immunosuppressive signaling and restore antitumor immune activity. In atherosclerosis, by contrast, translational challenges center on plaque access, foam-cell targeting, inflammatory control, and long-term safety during chronic administration. Cardiovascular payloads are usually directed toward reducing lipid-associated stress, limiting inflammatory amplification, and stabilizing vulnerable lesions. The two diseases share a macrophage-centered delivery logic but require distinct choices of targeting ligand, carrier architecture, release trigger, and therapeutic cargo.

**Figure 2:**
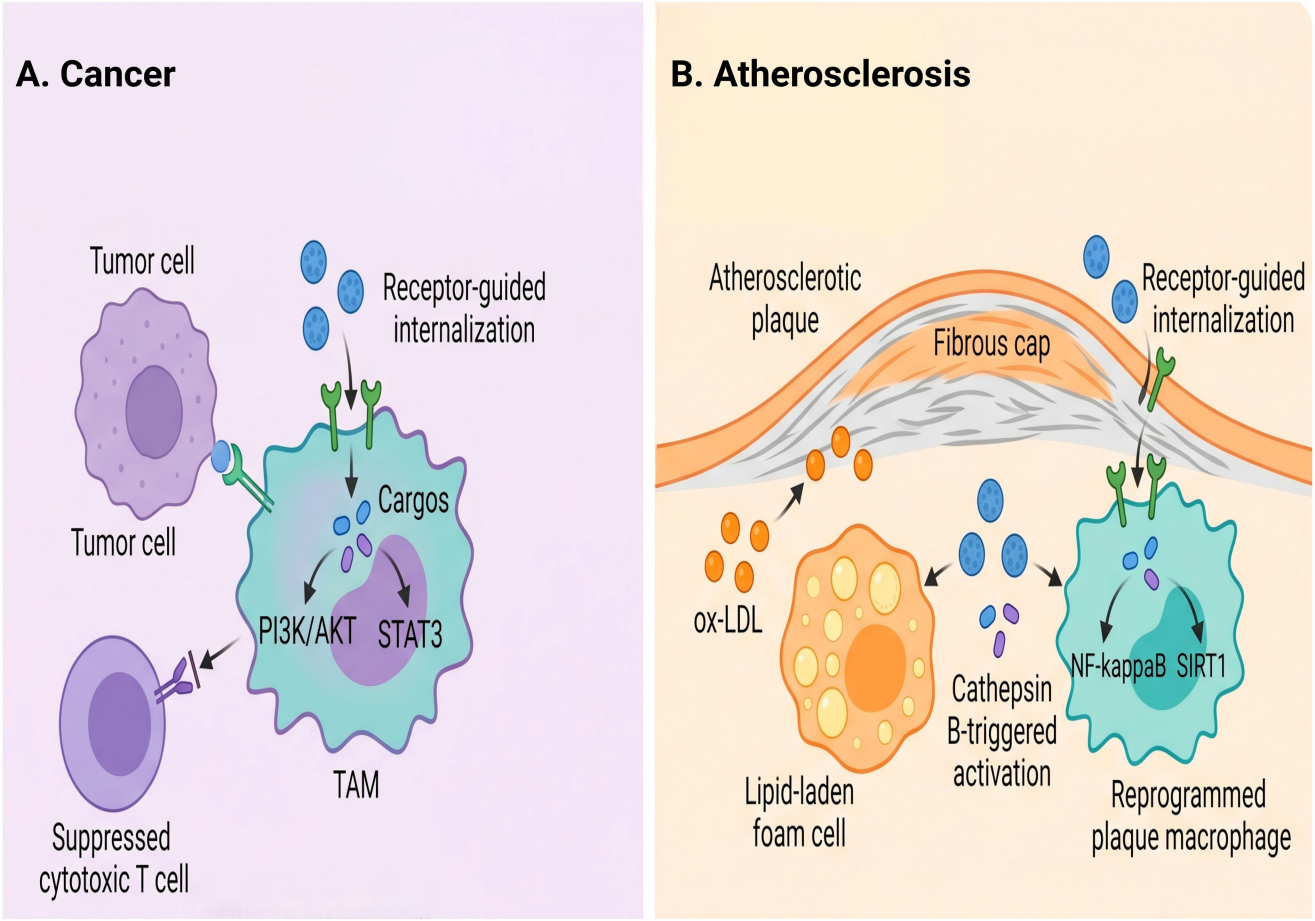
Disease-specific applications of macrophage-targeted nanodelivery in cancer and atherosclerosis. **(A)** In cancer, receptor-guided nanocarriers can be internalized by tumor-associated macrophages (TAMs) and release pathway-modulating cargos that target intracellular programs such as phosphoinositide 3-kinase/protein kinase B (PI3K/AKT) and signal transducer and activator of transcription 3 (STAT3). These interventions may reduce TAM-associated immunosuppressive functions, support TAM reprogramming, and improve antitumor immune activity. **(B)** In atherosclerosis, receptor-guided nanocarriers can enter plaque macrophages and foam cells within atherosclerotic lesions. Cathepsin B-triggered activation and intracellular cargo release may modulate inflammatory and metabolic pathways, including nuclear factor-kappa B (NF-kappaB) and sirtuin 1 (SIRT1), thereby reducing lipid-associated stress, limiting inflammatory amplification, and supporting plaque macrophage remodeling. ox-LDL, oxidized low-density lipoprotein; NF-kappaB, nuclear factor-kappa B; PI3K/AKT, phosphoinositide 3-kinase/protein kinase B; SIRT1, sirtuin 1; STAT3, signal transducer and activator of transcription 3; TAMs, tumor-associated macrophages. Created in BioRender. Huang, (Y) (2026) https://BioRender.com/9gjiea6.

Within this disease-specific framework, strategies can be interpreted according to the intracellular programs. Some platforms target kinase pathways involved in macrophage survival and polarization, such as PI3K/AKT-related signaling (42, 88). Others interfere with transcriptional programs that stabilize pathological macrophage states, including STAT3-centered signaling (40, 76, 88). In atherosclerosis, additional strategies should focus on correcting lipid-associated metabolic stress (36) or dampening chronic inflammatory signaling (32). The disease-specific applications of macrophage-targeted nanodelivery in cancer and atherosclerosis are summarized in **Figure 2**.

### 5.1 Cancer: reprogramming TAMs through mechanism-aligned intracellular interventions

TAMs constitute one of the major immune cell populations in many solid tumors (7) and often acquire immunosuppressive, tissue-remodeling, and M2-like functional features (138, 139). These cells promote tumor progression through extracellular matrix remodeling, angiogenesis, suppression of cytotoxic T-cell activity, and therapy resistance (7, 140). In this setting, macrophage-targeted nanomedicine is most effective when it does more than simply enter TAMs; it also engages intracellular pathways that sustain their tumor-supportive phenotype (42).

One important translational strategy targets signaling pathways that support macrophage survival and M2-like stabilization (42, 88). A representative case is the raspberry-like GD@PP/Wtmn micellar system (141), in which gemcitabine-conjugated dendritic poly-L-lysine nanoparticles were linked to wortmannin-loaded polymeric micelles through a CTSB-cleavable peptide (141). Receptor-guided macrophage uptake is coupled with CTSB-cleavable intracellular release (141), allowing wortmannin to interfere with PI3K-associated signaling programs that support M2 polarization and immunosuppressive macrophage function (88, 141). In this design, wortmannin-mediated TAM reprogramming is combined with gemcitabine-mediated tumor-cell cytotoxicity, weakening microenvironmental tumor support while preserving direct anti-tumor activity (141). This example illustrates how targeting PI3K-related macrophage polarization programs can convert the TME into a more treatment-responsive state (141, 142).

Another strategy is to disrupt transcriptional programs that stabilize TAM-associated immunosuppressive states (39, 77). STAT3 is particularly relevant in this context because it links cytokine-rich TME to durable macrophage transcriptional remodeling (40). For example, cluster of differentiation 163 (CD163)-targeted corosolic acid-containing long-circulating liposomes were developed to inhibit STAT3 specifically in CD163-positive monocytes and macrophages, providing a direct example of receptor-guided STAT3 modulation in macrophage-rich tumor settings (121, 143). Macrophage-targeted liposomes and endocytosis-compatible lipid nanoparticles can deliver STAT3-modulating cargos to TAMs or tumor-associated suppressive immune cells (40, 42). More recent RNA interference (RNAi)-based approaches have also explored systemic silencing of STAT3, alone or together with immune checkpoint-related targets such as programmed death ligand 1 (PD-L1), in tumor-associated suppressive immune cells (40, 77, 144). After intracellular release, such formulations can reduce STAT3 activation, attenuate M2-associated marker expression, and promote a more inflammatory macrophage phenotype (39, 76). In some models, this effect is accompanied by increased recruitment or activation of anti-tumor immune cells, suggesting that the therapeutic value of STAT3-targeted nanotherapy extends beyond macrophage-intrinsic signaling to broader immune microenvironment remodeling (77, 144). Together, these studies support STAT3 modulation as a practical route for converting selective TAM delivery into functional immune reprogramming (39).

Stress-adaptive pathways provide distinct molecular entry points for TAM reprogramming. The unfolded protein response (UPR), particularly protein kinase R-like endoplasmic reticulum kinase (PERK)-associated signaling, has been implicated in the metabolic and immunosuppressive functions of macrophages (145). This suggests that endoplasmic reticulum stress adaptation may help stabilize suppressive TAM-like states. Consistent with this concept, siRNA-loaded magnetic nanoparticles have been developed to modulate UPR-related signaling in a TAM-like experimental model, supporting the potential of targeting stress-response pathways for macrophage reprogramming (146). These studies broaden the intracellular vulnerability network beyond STAT3 and PI3K/AKT, indicating that macrophage-directed nanomedicine can also exploit stress-adaptive programs to reshape TAM function.

TAM remodeling is a powerful therapeutic strategy, but its greatest translational value may emerge when integrated with broader anticancer regimens (28, 147, 148). This is particularly relevant in glioblastoma and other refractory tumors, where therapeutic delivery should overcome anatomical barriers and macrophage-rich immunosuppressive microenvironments (28, 149). A representative example is a CTSB-triggered programmed brain-targeted delivery system designed for simultaneous TAM-targeted and glioblastoma-targeted delivery (28). Such platforms illustrate how barrier penetration, macrophage selectivity, tumor-cell targeting, and conditional intracellular release can be integrated within one therapeutic design (28, 149). In these cases, the delivered cargos may include STAT3-directed nucleic acids (150), immune agonists (151), chemotherapeutics, or combination payloads that simultaneously modulate macrophage phenotype and tumor-associated immune activity (148, 152). Macrophage-centered nanomedicine can function as a therapeutic amplifier rather than merely as a stand-alone macrophage-targeted intervention (147). By aligning receptor-mediated uptake, intracellular release, and mechanism-matched TAM remodeling, such platforms may increase the effectiveness of chemotherapy, immune checkpoint blockade, or localized immunostimulation (28).

### 5.2 Atherosclerosis: correcting lipid-inflammatory macrophage states through metabolic and inflammatory network modulation

In atherosclerosis, pathological macrophage dysfunction is driven by an interlocking triad: lipid-induced metabolic stress, persistent inflammatory activation, and protease-mediated plaque destabilization (62, 153). Accordingly, nanomedical interventions are designed to dismantle these specific drivers through three corresponding goals: correcting metabolic imbalance, suppressing inflammatory signaling, and stabilizing vulnerable microenvironments (59, 137, 154). For instance, delivering the SIRT1 activator SRT1720 via anti-CD36-targeted mesoporous silica nanoparticles (62) directly links receptor-mediated entry to intracellular metabolic modulation. By selectively accumulating in CD36-high cells, this system may correct lipid-associated stress and attenuate the pathological foam-cell state (62). This case illustrates how receptor-guided delivery can be paired with metabolic modulation to correct foam-cell dysfunction (62).

Another approach focuses on suppressing inflammatory amplification circuits in plaque macrophages (61, 155). A representative strategy involves ligand-modified superparamagnetic iron oxide nanoparticles that deliver microRNA-146a (miR-146a) into atherosclerotic lesions (61). These formulations combine receptor-guided accumulation, nucleic acid delivery, and imaging potential (61). They dampen NF-kappaB-associated inflammatory outputs and reduce inflammatory reinforcement that contributes to lesion progression (61).

Additional plaque-focused strategies further illustrate the need to match therapeutic payloads to macrophage dysfunction in atherosclerosis. Organelle stress, impaired autophagy, and defective lipid handling are closely linked to inflammatory macrophage activation and foam-cell persistence within plaques. Nuclear receptor Dax1 has been reported to promote atherosclerosis by inhibiting macrophage lipid transport and suppressing autophagy, suggesting that lipid-handling and autophagy-related pathways may represent additional targets for metabolic correction in plaque macrophages (156). These findings broaden the therapeutic framework beyond lipid uptake and NF-kappaB signaling by highlighting intracellular stress-adaptive and autophagy-associated programs. Delivery of IRF5 siRNA has been shown to reprogram inflammatory plaque macrophages, restore defective efferocytosis, and enhance the clearance of apoptotic foam cells in atherosclerotic lesions (157).

Although CTSB-triggered nanomedicine is predominantly utilized in oncology, its design logic may also be relevant to vulnerable atherosclerotic plaques (59, 137). Unstable plaques often contain macrophage-rich regions characterized by inflammatory burden, lysosomal stress, elevated protease activity, and extracellular matrix remodeling, creating a plausible microenvironment for protease-gated drug release (59, 158). This enzymatic trigger could be used to deliver anti-inflammatory agents, autophagy modulators, or imaging probes into protease-active plaque macrophages (32, 59, 137, 158). However, direct plaque-focused evidence remains limited, so this strategy should be viewed as an emerging extension of macrophage-targeted nanomedicine rather than an established therapeutic platform (59, 137).

Representative mechanism-aligned strategies for macrophage reprogramming in cancer and atherosclerosis are summarized in **Table 1**.

**Table 1:** Representative mechanism-aligned nanodelivery strategies for macrophage reprogramming in cancer and atherosclerosis.

Disease context	Targeted macrophage population	Release strategy	Intracellular network	Representative platform	Main translational implication
Pancreatic Cancer/TAM-rich stromal tumors	TAMs in the tumor microenvironment	Cathepsin B-cleavable release	PI3K/AKT-linked polarization control	Gemcitabine/wortmannin co-loaded raspberry-like micelles (141)	TAM remodeling combined with tumor-cell cytotoxicity
TAM-rich solid tumors/Macrophage-rich tumor models	Immunosuppressive TAMs or myeloid cells	Formulation-dependent intracellular release	STAT3-centered transcriptional stabilization	STAT3 inhibitor- or STAT3 siRNA-loaded nanocarriers (40, 76, 88, 121, 143, 144)	Reduced suppressive signaling and enhanced inflammatory reprogramming
Glioblastoma	TAMs/microglia in barrier-protected tumors	Protease-responsive or gated release	Integrated immune-reprogramming networks	Biomimetic or dual-targeted systems delivering STAT3-directed cargos or immune agonists (28, 147–150)	Improved macrophage remodeling and chemo-immunotherapy responsiveness
Atherosclerosis	CD36-high foam cells	Receptor-guided intracellular delivery	Metabolic correction/SIRT1-related regulation	Anti-CD36 mesoporous silica nanoparticles loaded with SRT1720 (62)	Reduced lipid-associated foam cell dysfunction
Atherosclerosis	Plaque macrophages	Intracellular release after lesion accumulation	NF-kappaB-associated inflammatory signaling	Ligand-modified miR-146a nanoparticles or superparamagnetic iron oxide nanoparticle (SPION)-based systems (61)	Dampened plaque inflammation and improved macrophage stability
Protease-active inflammatory lesions	Macrophages in protease-rich plaques or lesions	Cathepsin B-triggered or multi-trigger release	Protease-linked intracellular modulation	Cathepsin B-triggered or multi-responsive plaque-targeted nanocarriers (32, 59, 137, 154)	Emerging strategy for lesion-selective activation

This table summarizes selected macrophage-directed nanomedicine strategies according to disease context, targeted macrophage population, release strategy, intracellular network engagement, representative platform, and translational implication. Across these examples, therapeutic benefit depends not only on macrophage internalization, but also on the alignment between uptake route, intracellular release mechanism, and disease-relevant pathway modulation.

## 6 Translational challenges and limitations

Macrophage-targeted nanomedicine offers a promising strategy for reprogramming pathological macrophages, but its clinical translation remains limited by several design-specific barriers (16, 159, 160). Key limitations include target heterogeneity, off-target uptake, variable trigger responsiveness, intracellular delivery barriers, long-term biosafety, and scalable manufacturing (16, 42, 88, 159–162). These issues are especially relevant to receptor-guided internalization and CTSB-triggered release, which require lesion accumulation, selective receptor engagement, reliable intracellular cleavage, and biologically active payload delivery (16, 42).

### 6.1 Off-target uptake and long-term biosafety

One major translational barrier is the limited specificity of macrophage-targeted delivery (160, 163, 164). Scavenger receptors are not uniquely expressed on pathological macrophages; they are also present on resident macrophages in the liver, spleen, lung, and other organs involved in immune surveillance and physiological clearance (160, 165). Nanoparticles may be sequestered by the mononuclear phagocyte system (MPS) before reaching the intended lesion (163, 166). This can reduce drug accumulation at the disease site while increasing unintended exposure in non-target tissues (164). Protein corona formation further complicates targeting specificity (167–169). After intravenous administration, nanoparticles rapidly adsorb serum proteins, which may alter surface charge, ligand accessibility, receptor recognition, and biodistribution (168, 170). Therefore, targeting behavior observed in simplified *in vitro* models may not be fully retained *in vivo* (167). Long-term biosafety is another concern, especially for multicomponent systems containing cleavable peptide linkers, synthetic polymers, inorganic cores, or biomimetic coatings (76, 161, 162, 171). These complex architectures may pose risks related to immunogenicity, altered macrophage function in non-target tissues, delayed degradation-product clearance, and cumulative organ burden after repeated administration (88, 162, 171–173). Biomimetic strategies may reduce premature clearance, but they also introduce additional uncertainty regarding long-term immune tolerance and off-target macrophage activation (167).

### 6.2 Target heterogeneity and variable trigger responsiveness

Target receptor expression and intracellular trigger activity are highly heterogeneous in pathological macrophages (19, 75). Macrophages within the same tumor or atherosclerotic plaque may differ in scavenger receptor abundance (19), lysosomal activity (125), metabolic state, and responsiveness to reprogramming stimuli. This spatial and functional diversity complicates the design of universal delivery strategies. More specifically, heterogeneous scavenger receptor expression may lead to uneven receptor-guided internalization (46, 47). Some macrophage subsets may be efficiently targeted, others remain poorly accessed (19, 30, 46).

CTSB activity shows similar variability (26). Although CTSB is frequently elevated in tumors, inflammatory lesions, and macrophage-rich pathological niches, its activity can still vary across disease stages, tissue compartments, and regions within the same lesion (26, 59, 91). Some lesions may exhibit strong protease activity, whereas others may have insufficient activity to efficiently cleave CTSB-sensitive linkers (27). In such cases, nanocarriers that rely on a single enzymatic trigger may show incomplete activation, heterogeneous drug release, or reduced therapeutic consistency (27, 91). These variations may uncouple receptor-mediated uptake from effective intracellular payload release, which is a central limitation of CTSB-triggered cleavage design (76, 174).

This variability highlights the need for biomarker-informed stratification (59, 91). Patients or lesions with high scavenger receptor expression and elevated protease activity may be more likely to benefit from receptor-guided and CTSB-triggered systems (19). CTSB-sensitive designs may not provide reliable activation when trigger activity is heterogeneous (27, 91). In such cases, additional pH-, ROS-, glutathione-, or externally responsive modules may be needed (27).

### 6.3 Intracellular delivery efficiency and functional bioavailability

Efficient cellular internalization does not necessarily translate into functional intracellular delivery (174). Even after receptor-guided internalization and CTSB-triggered cleavage, therapeutic cargos may fail to reach their effective intracellular sites of action (76). Different cargos face different intracellular barriers: small molecules may remain trapped in vesicles, nucleic acids may be degraded, and proteins or peptides may lose activity before reaching the cytoplasm (174). This problem is particularly relevant for payloads designed to regulate signaling complexes, transcriptional programs, or intracellular metabolism (14).

For this reason, macrophage-targeted nanotherapy should not be evaluated solely by cellular internalization or lesion accumulation (175). Functional bioavailability is equally important. Nanoparticles should be assessed for whether the released payload reaches sufficient cytosolic or nuclear concentrations in a biologically active form (76). Early-stage fluorescence-based internalization assays may overestimate therapeutic relevance if they do not demonstrate functional intracellular delivery and corresponding biological activity (31, 76, 175).

### 6.4 Manufacturing, standardization, and disease-relevant models

The pharmaceutical development of increasingly sophisticated nanocarriers constitutes a major translational bottleneck (176, 177). Although integrating multiple design elements may enhance biological precision, it also increases manufacturing complexity (71, 178). For successful clinical translation, nanocarriers must be reliably manufactured at scale with consistent batch-to-batch quality (177, 179). This is critical for hybrid platforms because subtle variations in surface ligand density may alter scavenger receptor engagement and compromise regulatory standardization (176, 178, 180). behavior The reliance on conventional preclinical models remains another important limitation (181). Standard macrophage cultures and short-term mouse models often fail to capture the chronic and structural complexity of human diseases (159). For instance, pancreatic stromal barriers and advanced atherosclerotic plaques may limit nanoparticle penetration and cellular access in ways that simplified models cannot reliably predict (182, 183).To bridge this gap, future preclinical evaluations should increasingly incorporate patient-derived organoids, microfluidic barrier systems, patient-derived tissue specimens, and disease-relevant *in vivo* platforms that better reflect clinical delivery constraints (181, 184).

Key translational challenges and future directions for macrophage-targeted nanomedicine are summarized in **Table 2**.

**Table 2:** Key translational challenges and future directions for macrophage-targeted nanomedicine.

Challenge	Translational concern	Suggested direction
Targeting specificity	Scavenger receptors are also expressed by resident macrophages in non-target organs (155–163).	Improve ligand design, use dual-targeting strategies, and assess off-target macrophage internalization.
Protein corona	Serum proteins may mask targeting ligands and alter biodistribution (163–166, 171).	Characterize corona formation and optimize surface shielding.
Receptor heterogeneity	Disease-associated macrophages show variable CD36, MSR1, MARCO, and SR-B1 expression (181, 182).	Use single-cell and spatial profiling for target selection and patient or lesion stratification.
Cathepsin B variability	Protease activity differs across lesions, disease stages, and macrophage states (94, 99, 107, 108).	Combine cathepsin B triggers with pH-, ROS-, glutathione-sensitive, or externally activated modules.
Intracellular bioavailability	Internalization may not lead to cytoplasmic or nuclear delivery (135, 172).	Evaluate release kinetics, endosomal escape, pathway inhibition, and phenotype conversion.
Payload selection	Cargo may not match the pathway maintaining the pathological macrophage phenotype (88, 130, 134, 137).	Align payloads with PI3K/AKT, STAT3, NF-kappaB, SIRT1, or metabolic vulnerabilities.
Long-term safety	Repeated dosing may affect non-target macrophages or cause cumulative organ burden (173, 174, 185).	Use biodegradable materials and perform long-term immunotoxicity studies.
Manufacturing	Complex formulations may lack reproducibility and scalable production (173, 175, 185).	Define critical quality attributes and simplify platform design when possible.
Disease models	Conventional models may not capture human macrophage heterogeneity or tissue barriers (177, 180–182).	Incorporate patient-derived, organoid, microfluidic, and disease-relevant in vivo models.

This table summarizes major barriers that may limit the clinical translation of scavenger receptor-guided and cathepsin B-triggered nanodelivery systems. Successful development will require improved targeting specificity, reliable trigger responsiveness, functional intracellular delivery, mechanism-matched payload selection, long-term safety evaluation, scalable manufacturing, and disease-relevant preclinical models.

## 7 Future perspectives and conclusion

Macrophage dysfunction is a defining feature of many solid tumors, where these cells orchestrate immune suppression, tissue remodeling, and therapeutic resistance (7, 140). In atherosclerosis, pathological macrophage states drive persistent inflammation and mediate plaque destabilization (62, 153). Their phenotypic plasticity makes them attractive therapeutic targets, but also poses a substantial challenge for precise intervention (2). In this context, nanomedicine offers a rational framework for macrophage-directed therapy because it enables the integration of selective internalization, intracellularly controlled release, and phenotype-modulating payload delivery (15, 137, 186).

A key priority for the field is the more precise characterization of disease-associated macrophage states. Advanced tools such as single-cell transcriptomics and spatial omics are critical for identifying which macrophage states drive pathogenesis and possess suitable receptors for targeted intervention (23, 76, 187). These approaches will clarify how targetable receptors, proteolytic triggers, and intracellular vulnerabilities spatially co-occur within clinically meaningful lesions (181, 188). Such mechanistic mapping is essential for selecting predictive disease models, optimizing nanocarrier design, and stratifying patients for targeted therapies (23, 189).

Nanocarrier design should evolve from simple lesion targeting toward mechanism-matched programming (39, 42). The goal is to synchronize receptor-guided internalization and protease-triggered release with the regulatory vulnerabilities of the target cell (137). Furthermore, integrating mechanistic biomarkers into clinical development may enable more precise patient stratification (36), transforming macrophage-targeted nanotherapy from a generalized delivery concept into a more personalized therapeutic strategy (190).

The therapeutic success of these systems is not guaranteed by delivery alone. It depends on how effectively the released payload disrupts intracellular vulnerability networks that maintain pathological macrophage states (38). STAT3 represents an important but context-dependent and translationally relevant node within this broader network, rather than an exclusive mechanistic axis (40, 77). Evidence from cancer and atherosclerosis supports the therapeutic promise of this approach (36, 137). However, successful translation will require improved targeting specificity, more reliable trigger responsiveness, greater intracellular bioavailability, and scalable formulation design (159). The future of this field may depend less on increasing carrier complexity alone and more on improving mechanistic coherence between targeting, intracellular processing, and biological outcome (162).
